# Anti-Rituximab Antibodies Occurrence and Clinical Outcomes in Patients With Primary Membranous Nephropathy

**DOI:** 10.1016/j.ekir.2025.04.059

**Published:** 2025-05-06

**Authors:** Marco Allinovi, Maxime Teisseyre, Matteo Accinno, Cecilia Finocchi, Vincent L.M. Esnault, Marion Cremoni, Tommaso Mazzierli, Daniela Lazzarini, Micaela Anna Casiraghi, Céline Fernandez, Kévin Zorzi, Vesna Brglez, Lorenzo Cosmi, Andrea Matucci, Leonardo Caroti, Giulia Antognoli, Calogero Lino Cirami, Alessandra Vultaggio, Barbara Seitz-Polski

**Affiliations:** 1 Nephrology, Dialysis and Transplantation Unit, Careggi University Hospital, Florence, Italy; 2 French Reference Center for Rare Diseases, Idiopathic Nephrotic Syndrome and Membranous Nephropathy, Nice University Hospital, Université Côte d’Azur, Nice, France; 3 Immunology Laboratory, Nice University Hospital, Université Côte d’Azur, Nice, France; 4 Department of Nephrology, Dialysis and Transplantation, Nice University Hospital, Université Côte d’Azur, Nice, France; 5 Institut de Recherche sur le Cancer et Vieillissement UMR7284 CNRS INSERM U1081, Université Côte d’Azur, Nice, France; 6 Department of Experimental and Clinical Medicine, University of Florence, Florence, Italy; 7 Immunoallergology Unit, Careggi University Hospital, Florence, Italy

**Keywords:** antidrug antibody, anti-rituximab antibody, immunogenicity, membranous nephropathy, neutralizing antibodies, rituximab

## Abstract

**Introduction:**

Rituximab is a first-line treatment for primary membranous nephropathy (pMN), with proven efficacy and safety. The use of monoclonal antibodies such as rituximab can lead to the formation of antidrug antibodies that may interfere with the therapeutic response. In pMN, anti-rituximab antibodies (ARAs) have been shown to neutralize the cytotoxicity of rituximab, thereby increasing the risk of relapse of nephrotic syndrome. However, the kinetics of ARAs over time and the effect of ARA titer on prognosis are unclear.

**Methods:**

This retrospective international multicenter study included 74 patients with pMN treated with rituximab. Here we aimed to clarify the correlation between ARAs and clinical outcome, as well as to evaluate the most appropriate timing of ARA detection.

**Results:**

Overall, 35 out of 74 patients (47%) developed ARAs after a median of 9 (interquartile range: 6–12) months following rituximab administration. ARA monitoring at month-9, month-12 and before rituximab readministration identified 88% of patients with ARAs. Clinical remission rate at 6 and 12 months after rituximab administration was significantly lower in patients with ARAs (31% vs. 56%, *P* = 0.03 and 54% vs. 87%, *P* = 0.0017, respectively). ARAs were associated with a significantly higher rate of relapse (63% vs. 29%, *P* = 0.036) and a higher rate of B-cell reconstitution at 6 months (74.2% vs. 50%, *P* = 0.048). Notably, relapse occurred earlier in patients with ARAs (22 months vs. 32 months, *P* = 0.01).

**Conclusion:**

The development of ARAs represents one of the most important prognostic factors in pMN, being significantly associated with a reduced remission rate and a higher relapse rate after rituximab therapy. Alternative therapies with obinutuzumab or ofatumumab should be considered for these patients.

## Graphical abstract

**Figure:**
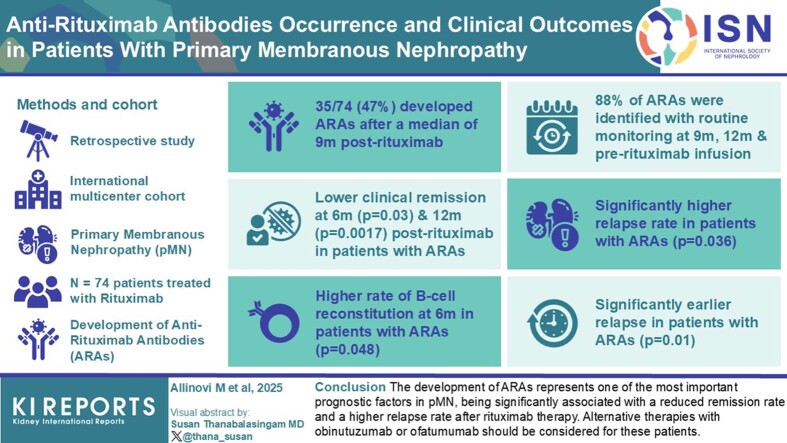

See Commentary on Page 2545

Membranous nephropathy is the most common cause of nephrotic syndrome in nondiabetic adults worldwide. Its incidence ranges from 8 to 10 cases per 1 million population, of which approximately 80% are pMN.^1^ Primary forms are autoimmune-mediated glomerular diseases characterized by immune system dysregulation leading to podocyte damage through autoantibodies and complement system activation.^2^, ^3^, ^4^ Autoantibodies directed against the M-type phospholipase A2 receptor (anti-PLA2R) are the most common.^2^^,^^5^, ^6^, ^7^ The identification of the membranous nephropathy as autoimmunity-related glomerulonephritis, characterized by the presence of autoantibodies, has promoted the use of immunosuppressive therapies.^8^^,^^9^ According to the Kidney Disease: Improving Global Outcomes guidelines, rituximab represents a first-line therapy for the management of pMN in patients at moderate or high risk, with high efficacy in inducing remission while remaining safe.^10^

However, in some patients, rituximab is ineffective or becomes ineffective after repeated administrations.^11^ Three main factors may interfere with the efficacy of rituximab in pMN as follows: (i) the presence of chronic glomerular damage, in which immunosuppressive treatment is not effective and which requires optimization of supportive care; (ii) urinary drug loss, which can lead to underdosing and the need for repeat treatment^12^, ^13^, ^14^; and (iii) the presence of ARAs.^14^^,^^15^

Indeed, the use of monoclonal antibodies such as rituximab can lead to the formation of antidrug antibodies that may interfere with the therapeutic response.^15^, ^16^, ^17^ Approximately 23% to 43% of patients with pMN treated with rituximab will develop ARAs.^14^^,^^17^ ARAs are detected after a mean interval of 9 months following rituximab administration.^18^ Different factors may promote their formation.^16^^,^^18^ Recent studies have shown that ARAs could reduce the efficacy of rituximab, impairing B-cell depletion and leading to a higher relapse rate in pMN and other immune-mediated diseases.^12^^,^^17^, ^18^, ^19^, ^20^ In systemic lupus erythematosus, it has been shown that 37.8% of patients developed ARAs after receiving rituximab.^21^ In this study, patients with ARAs were significantly younger, had more active disease, and had higher CD19+ counts at month 6 than patients without ARAs. In another study, 64.3% of patients with systemic lupus erythematosus treated with rituximab developed persistent ARAs.^22^ Patients with ARAs had lower drug exposure and relapsed earlier. ARAs showed neutralizing activity, suggesting that rituximab clearance by ARAs may contribute to relapse. In multiple sclerosis, ARAs were detected in 37% of relapsing-remission forms and 26% of progressive forms and were associated with incomplete B-cell depletion.^23^ In steroid-dependent pediatric nephrotic syndrome, ARAs were detected in 29% to 38% of patients and were associated with lower B-cell depletion and even a higher risk of relapse.^20^^,^^24^ ARAs also appear to be associated with the risk of developing adverse drug reactions following rituximab administration.^20^^,^^25^^,^^26^

As a result, the treatment of patients who have developed ARAs is a major challenge. In a previous study, we showed that obinutuzumab or ofatumumab was more effective than rituximab in patients with pMN and ARAs.^27^

Currently, the kinetics of ARAs over time and the impact of ARA titer on prognosis are poorly understood. In our study, we aimed to clarify the correlation between ARAs and clinical outcome, as well as to assess the clinical relevance of their titer and the most appropriate timing of detection in pMN patients treated with rituximab.

## Methods

### Patients and Sampling

In this multicenter international study, we prospectively observed all consecutive patients scheduled for rituximab administration at the Careggi University Hospital, Florence, Italy and at Nice University Hospital, Nice, France.

The inclusion criteria were as follows: (i) aged > 18 years, (ii) membranous nephropathy diagnosed by renal biopsy or serological assay, and (iii) ARA monitoring within 12 months of rituximab treatment. The exclusion criteria were as follows: (i) concomitant immunosuppressive therapy other than rituximab; (ii) secondary membranous nephropathy defined by the presence of concomitant autoimmune disease (e.g., systemic lupus erythematosus); positive HIV, hepatitis B, or hepatitis C serologies; a positive cancer workup; or the use of medications such as COX-2 inhibitors, antitumor necrosis factor agents, lithium, or mercury-based treatments.

Patients were treated with rituximab according to the Kidney Disease: Improving Global Outcomes guidelines. All patients received a nonimmunosuppressive antiproteinuric treatment at the maximum tolerated dose containing a renin-angiotensin aldosterone system inhibitor and/or a diuretic combined with a low-salt diet. The clinical and laboratory parameters for all patients at baseline and then every 3 months after rituximab therapy were evaluated, such as 24-hour urinary protein excretion or urinary protein-to-creatinine ratio, serum albumin, serum creatinine, estimated glomerular filtration rate using the Chronic Kidney Disease-Epidemiology Collaboration formula, anti-PLA2R titer, ARA titer, and CD19+ cell count.

Forty-six patients had previously been reported in the paper by Teisseyre *et al.*,^18^ which mainly focused on the risk factors associated with the occurrence of ARAs; their follow-up has been extended by a median of 23 months.

### Definitions

Complete remission was defined as proteinuria < 0.3 g/24 h or 0.3 g/g in at least 2 consecutive visits. Partial remission was defined as proteinuria < 3.5 g/24 h or < 3.5 g/g and least 50% reduction versus baseline values. Immunological remission was defined as anti-PLA2R antibody titer < 14 AU/ml using enzyme-linked immunosorbent assay. Complete B cell depletion was defined as a CD19+ cell count ≤ 5/mm^3^. A relapse of the disease was defined as an increase in proteinuria > 3.5 g/24 h or > 3.5 g/g after achievement of clinical remission.

### Rituximab Regimen

Rituximab administration was standardized, with two 1000 mg infusions 2 weeks apart, except for 6 patients with four 375 mg/m^2^ rituximab infusions at 1 week intervals. Rituximab infusions were preceded by premedication (30 minutes before rituximab), based on i.v. infusion of hydrocortisone 100 mg or solumedrol 80 mg, chlorphenamine maleate 5 mg or 10 mg, and paracetamol 1000 mg.

### ARA Detection

ARAs were detected by using a commercially available bridging format enzyme-linked immunosorbent assay kit (LISA-TRACKER Duo Rituximab, Theradiag, Croissy Beaubourg, France). According to the manufacturer's instructions, sera were diluted 1:1000 in dilution buffer before the assay. The positivity cutoff limit of detection, as defined by the manufacturer, was 5 ng/ml.

ARAs were retrospectively monitored in serum samples collected longitudinally over at least 12 months as follows: at baseline (before rituximab treatment) and at 3, 6, 9, and 12 months after rituximab administration.

### Statistical Analysis

Categorical variables were described as frequencies and percentages and were analyzed using chi-square test or Fisher exact test. A *P* < 0.05 was considered statistically significant. Continuous variables were analyzed using a 2-tailed *t* test or the Mann-Whitney U test, as appropriate. Correlations were analyzed using the Spearman’s rank correlation test. Survival curves were assessed by the Kaplan-Meier method and compared with the log-rank test. Statistical analysis was performed using the SPSS 22.0 software package (IBM, Armonk, NY).

### Study Approval

The study protocol conformed to the ethical guidelines of the 1975 Declaration of Helsinki and was approved by the appropriate institutional review committee of different centers (25964_bio; NCT04326218 and NCT02199145). Written informed consent was obtained from each subject involved in the study.

## Results

### ARA Development in Patients With pMN Treated With Rituximab

ARAs were monitored in 74 patients with pMN treated with rituximab. The diagnosis of pMN was confirmed histologically in 64 patients (86%), and in the remaining patients by a positive serological test. Overall, 35 out of 74 patients (47%) developed ARAs. In Table 1, we describe the baseline characteristics of the patients. Patients who developed ARAs (ARA+) had a significantly higher anti-PLA2R1 antibody titer and body mass index than patients who did not develop ARAs (ARA−), and ARA+ patients tended to be older than ARA− patients (Table 1).

**Table 1:** Main characteristics of the patients with primary membranous nephropathy included in the study, in relation to anti-rituximab antibody positivity

Baseline	Overall (*N* = 74)	ARA+ (*n* = 35)	ARA− (n = 39)	*P*-*value*
Gender (Male), *n* (%)	49/74 (66.2%)	20/35 (57.1%)	29/39 (74.4%)	0.19
Age (yrs), median (Q1–Q3)	62.0 (51.0–72.0)	67.0 (53.5–73.5)	60.5 (51.0–73.0)	0.05
Creatinine (mg/dl), median (Q1–Q3)	1.25 (0.92–1.70)	1.28 (0.96–1.85)	1.22 (0.89–1.69)	0.38
eGFR (ml/min per 1.73 m^2^), median (Q1–Q3)	58 (39–88)	57 (34–83)	62 (34–88)	0.25
eGFR < 60 ml/min per 1.73 m^2^, *n* (%)	37/74 (50.0%)	18/35 (51.4%)	19/39 (48.7%)	0.82
Albumin (g/dl), median (Q1–Q3)	25.0 (20.0–30.0)	25.0 (20.0–29.9)	26.2 (20.8–30.0)	0.45
Proteinuria (g/24 h or g/g), median (Q1–Q3)	5.9 (4.2–7.8)	6.4 (4.6–7.8)	5.8 (4.3–7.7)	0.38
Naive for rituximab, *n* (%)	57/74 (77.0%)	26/35 (74.3%)	31/39 (79.5%)	0.60
Diagnosis > 5 years, *n* (%)	49/74 (66.2%)	24/35 (68.6%)	25/39 (64.1%)	0.68
Anti-PLA2R antibody positivity (%)	65/74 (87.8%)	30/35 (85.7%)	35/39 (89.7%)	0.86
Anti-PLA2R antibody titer (AU/ml), median (Q1–Q3)	84.6 (30.8–218.5)	167.0 (69.5–257.8)	70.5 (20.0–154.0)	0.029
Weight (kg), median (Q1–Q3)	74.0 (66.0–83.3)	76.6 (69.5–92.0)	71.6 (63.8–79.5)	0.109
BMI (kg/m^2^), median (Q1–Q3)	25.5 (23.7–28.2)	27.0 (24.0–30.3)	25.1 (22.9–27.4)	0.022
Outcomes				
B-cell reconstitution at 6 months, *n* (%)	39/63 (61.9%)	23/31 (74.2%)	16/32 (50.0%)	0.048
Immunological remission (anti-PLA2R < 14 AU/ml) at 6 mos, *n* (%)	41/61 (67.2%)	16/26 (61.5%)	25/35 (71.4%)	0.59
Stringent immunological remission (anti-PLA2R < 2 AU/mL) at 6 mos, *n* (%)	28/61 (46%)	10/26 (38.5%)	18/35 (51.4%)	0.32
Immunological remission (anti-PLA2R < 14 AU/ml) at 12 mos, *n* (%)	44/62 (71%)	18/28 (64.3%)	26/34 (76.5%)	0.44
Stringent immunological remission (anti-PLA2R < 2 AU/ml) at 12 mos, *n* (%)	32/62 (52%)	12/28 (43%)	20/34 (59%)	0.21
Clinical remission (CR + PR) at 6 mos, *n* (%)	33/74 (44.6%)	11/35 (31.4%)	22/39 (56.4%)	0.03
Clinical remission (CR + PR) at 12 mos, *n* (%)	53/74 (71.6%)	19/35 (54.3%)	34/39 (87.2%)	0.0017
Relapse rate at last follow-up, *n* (%)	22/53 (41.5%)	12/19 (63.2%)	10/34 (29.4%)	0.017
ADR, *n* (%)	11/74 (14.9%)	7/35 (20.0%)	4/39 (10.3%)	0.33
Infection-related ADR, *n* (%)	5/11 (45.5%)	4/7 (57.1%)	1/4 (25.0%)	0.55
Length of follow-up, median (mos) (Q1–Q3)	26.0 (16.5–56.0)	25.0 (16.5–49.5)	26 (16.5–60.0)	0.87

ADR, adverse drug reaction; ARA, anti-rituximab antibodies; ARA+, patients with anti-rituximab antibodies; ARA−, patients without anti-rituximab antibodies; BMI, body mass index; CR, complete remission; eGFR, estimated glomerular filtration rate; PR, partial remission; PLA2R, phospholipase A2 receptor; Q1, first quartile 1; Q3, third quartile.

**Figure 1:**
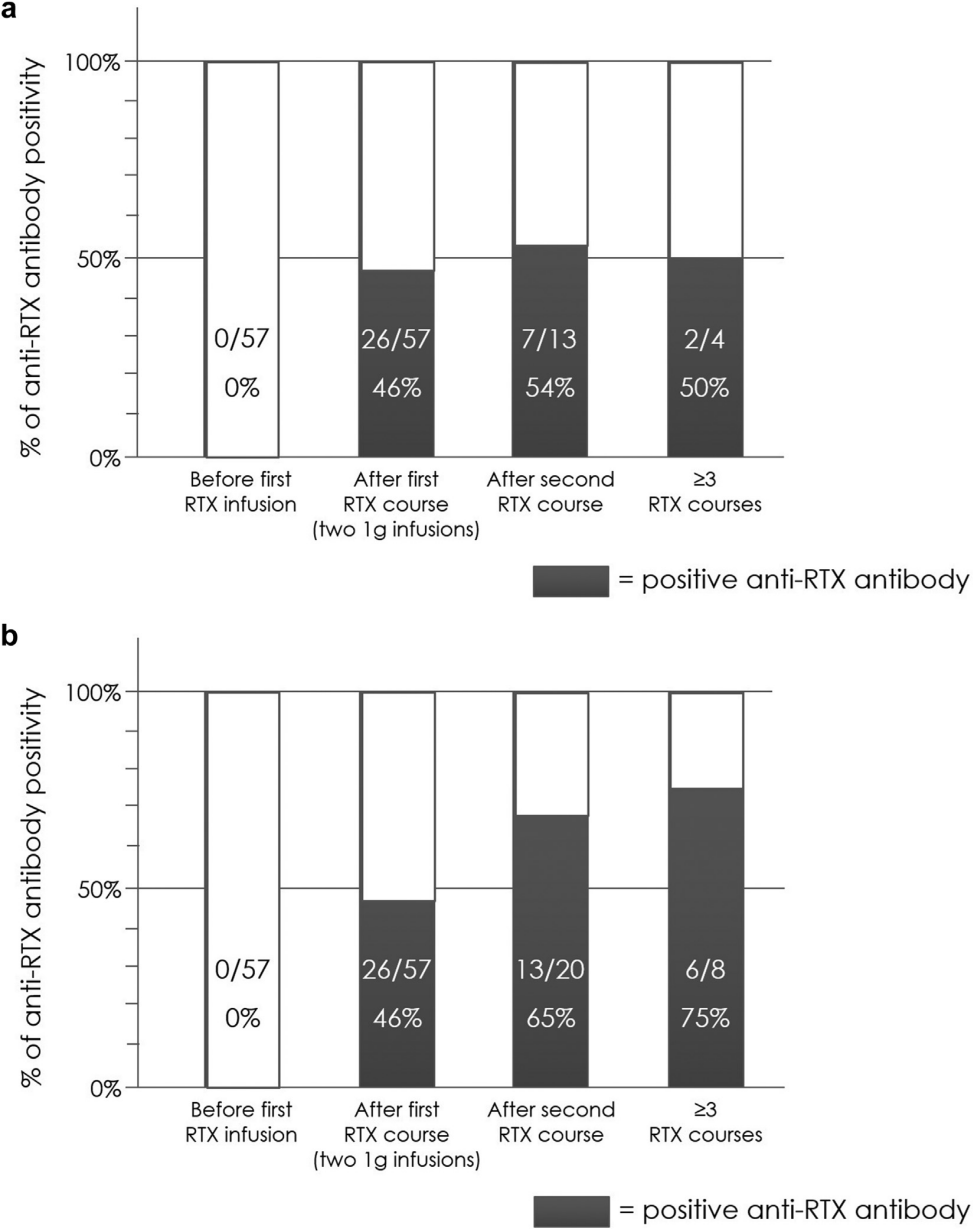
(a) Rate of anti-rituximab antibody positivity in 74 patients with membranous nephropathy and (b) rate of anti-rituximab antibody positivity in 85 rituximab courses from 74 individual patients. The rate of anti-rituximab antibody positivity increases progressively with exposure to subsequent rituximab courses.

We divided the 74 patients into 2 groups as follows: the first group (rituximab-naive patients) included patients studied starting from their first cycle of therapy (*n* = 57), whereas the second group (non–rituximab-naive patients) included patients who restarted a new cycle of therapy (*n* = 17) because of resistance to a previous rituximab or relapse. In the non–rituximab-naive group, patients were included after a median time of 26.5 months (range: 6.0–86.0) following the first rituximab infusion; monitoring of ARAs before the current course of rituximab was not available. Among the patients with available ARA monitoring at baseline, none (0/57) of rituximab-naive patients (0%) showed preexisting ARAs before starting rituximab therapy, 9 of 12 non–rituximab-naive patients (75%) had preexisting ARAs before the new rituximab treatment, whereas 3 of 12 non–rituximab-naive patients (25%) developed ARAs for the first time after the second rituximab course. Twenty-six out of 57 patients (46%) of the first group were ARA+; whereas 9 out of 17 patients (53%) of the second group were ARA+ (Figure 1a), of whom 7 were after the second course and 2 were after the third rituximab course.

In Figure 1b, we did not consider only individual patients, but included additional available ARA assessments for 11 patients who underwent subsequent rituximab courses with ARA monitoring. Considering 85 rituximab courses from 74 individual patients, we found that the rate of ARA positivity increases progressively with exposure to subsequent rituximab courses (Figure 1b).

### Kinetics of ARA Development

**Figure 2:**
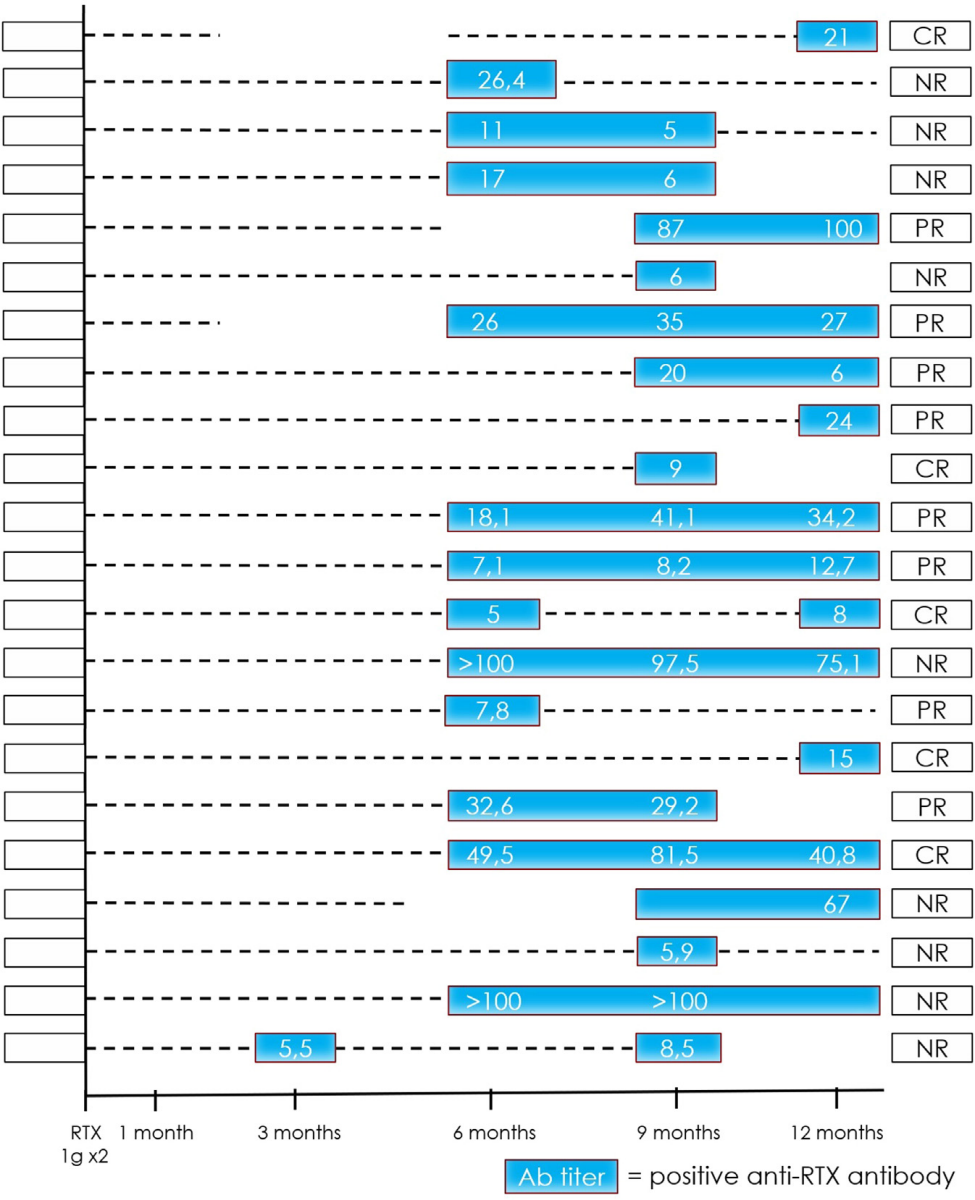
Anti-rituximab antibody titer from baseline to 18 months after rituximab infusion in rituximab-naive patients. The dotted line represents ARA negativity, whereas the blue rectangle represents ARA positivity at different time points with the ARA titer specified inside (when available). We considered only those patients with at least 3 out of five 3-monthly ARA assessments. ARA, anti-rituximab antibody; CR, complete remission; NR, no remission; PR, partial remission.

**Figure 3:**
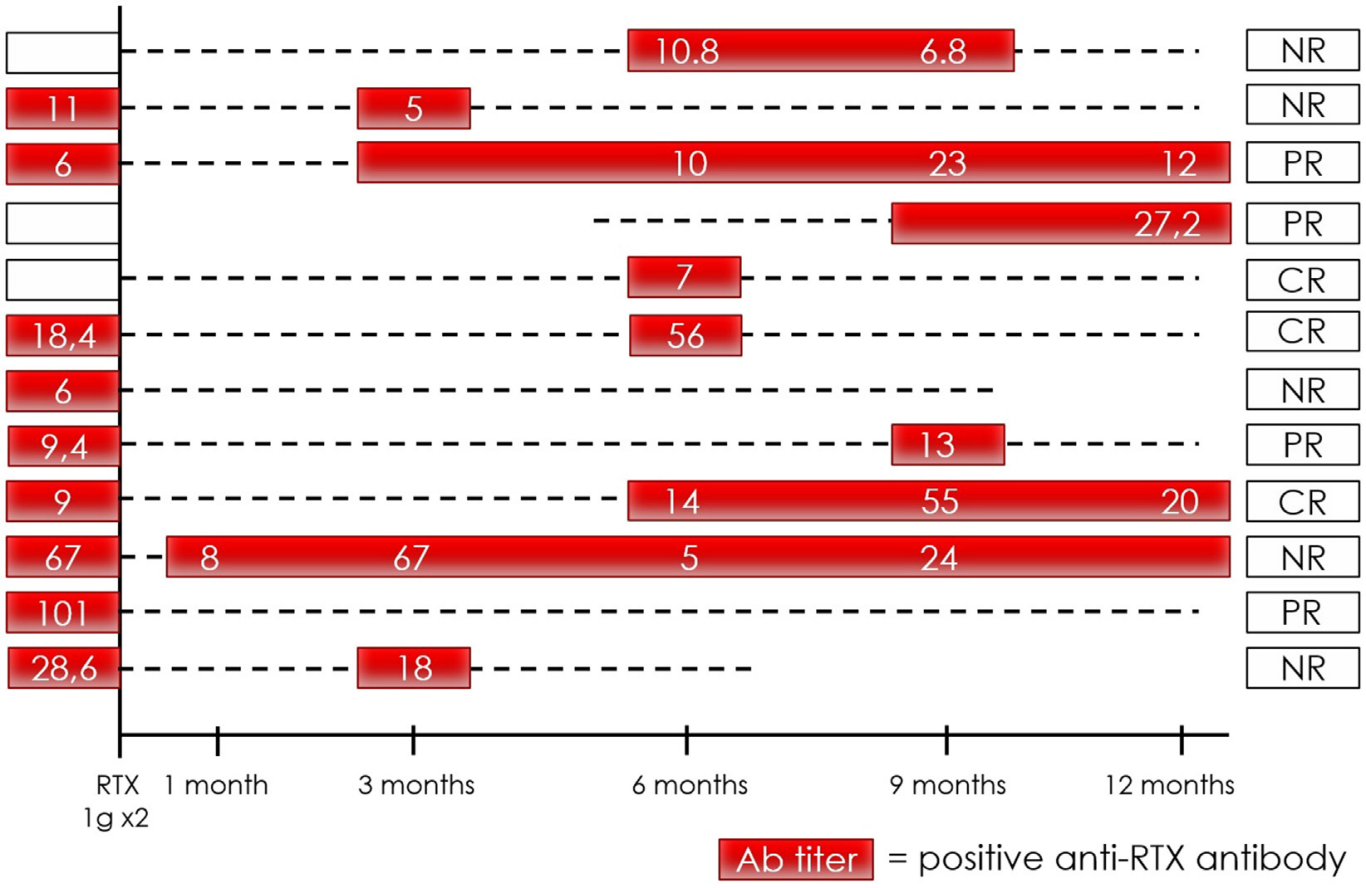
Anti-rituximab antibody titer from baseline to 18 months after rituximab infusion in non–rituximab-naive patients. The dotted line represents ARA negativity, whereas the red rectangle represents ARA positivity at different timepoints with the ARA titer specified inside (when available). We considered only those patients with at least 3 out of five 3-monthly ARA assessments ARA, anti-rituximab antibody; CR, complete remission; NR, no remission; PR, partial remission.

We then evaluated the kinetics of ARAs over time, both in rituximab-naive and non–rituximab-naive patients. Overall, ARAs were detected at a median of 9 (interquartile range: 6–12) months after rituximab administration. Monitoring ARA at 3, 6, 9, and 12 months after the treatment identified ARA+ patients in 16% (5/31), 56% (18/32), 67% (22/33), and 64% (18/28) of cases, respectively (Figures 2 and 3). Monitoring of ARA before rituximab readministration identified ARA+ patients in 6 of 9 cases (67%). Consequently, considering only patients who had available ARA monitoring at month 9 plus month 12 plus pre-rituximab, a “3 monitoring time points” approach, allowed the identification of 30 of 34 ARA+ patients (88%).

### Kinetics of ARA Titer

**Figure 4:**
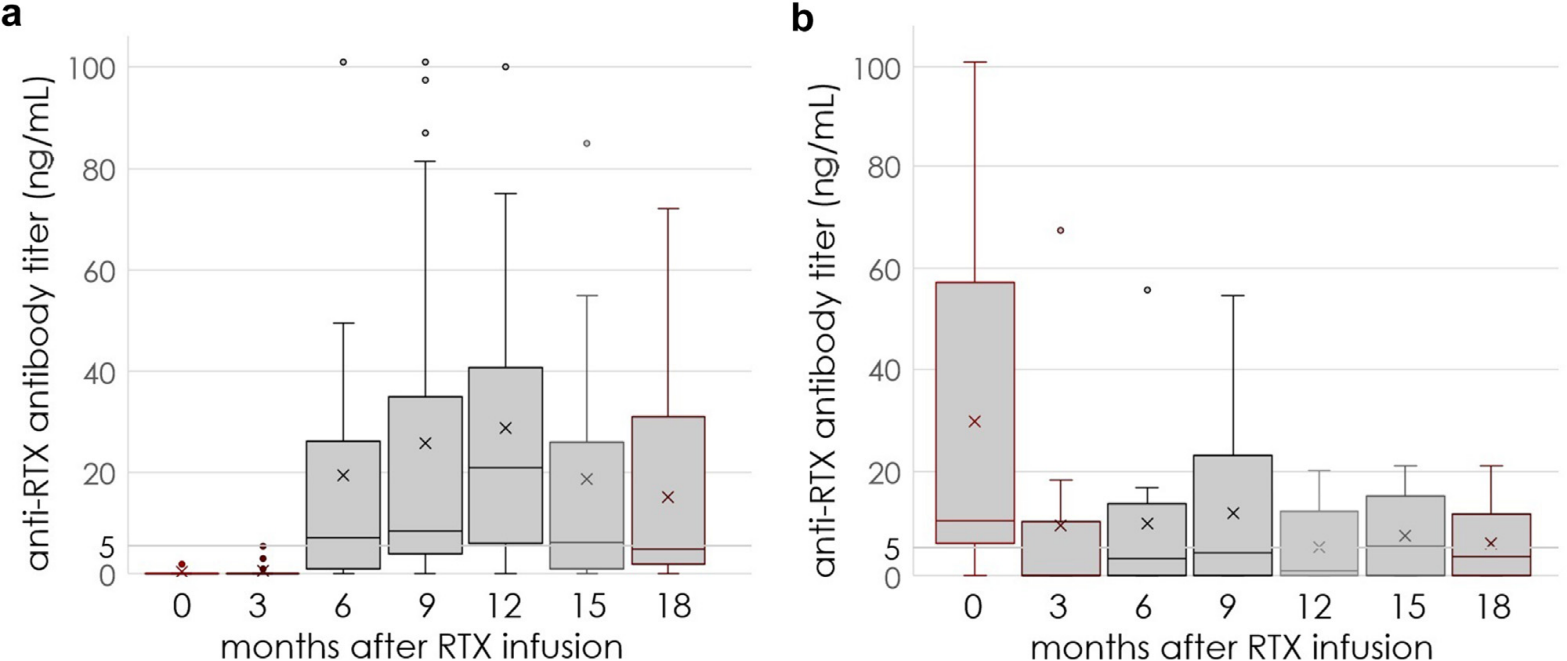
Trend in median anti-rituximab antibody titer from baseline to 18 months after rituximab infusion. Box plot showing the median anti-rituximab antibody titer, both in (a) rituximab-naive patients and (b) non–rituximab-naive patients.

In rituximab-naive patients with pMN who became ARA+, ARAs were not detected until month 6, after which the median ARA titer increased progressively, reaching a peak at 12 months, before decreasing progressively over the months following that (Figures 2 and 4a). Differently, in non–rituximab-naive patients who have previously developed ARAs before restarting treatment with rituximab, the median ARA titer became negative after rituximab infusion in all patients, and reappeared with variable delay (> 3 months in almost all patients), reaching a peak at 9 months, and then progressively decreasing over the months following (Figures 3 and 4b). Among the patients with available ARA monitoring at baseline, tracking the values for individual patients, it appeared that the ARA titer was not stable over time and only in 2 of 34 patients (6%), both rituximab-naive, the titer was still increasing at 12 months post-rituximab.

### Impact of ARAs Development on Clinical Outcomes in Patients With pMN

First, we investigated the clinical impact of ARA development on immunological remission rate. Overall, immunological remission rate at 6 and 12 months after rituximab administration was lower in patients with ARA+ than in without ARA− (16/26 [61.5%] vs. 25/35 [71.4%], *P* = 0.59 and 18/28 [64.3%] vs. 26/34 [76.5%], *P* = 0.44, respectively) although it did not reach statistical significance. Patients who were ARA+ had more frequent B-cell reconstitution at 6 months than patients who were ARA− (74.2% vs. 50%, *P* = 0.048).

Afterward, we investigated the clinical impact of ARA development on clinical remission rate. Overall, clinical remission rate at 6 and 12 months after rituximab administration was significantly lower in patients who were ARA+ than in ARA− (11/35 [31%] vs. 22/39 [56%], *P* = 0.03 and 19/35 [54%] vs. 34/39 [87%], *P* = 0.0017, respectively). Considering only patients who were rituximab-naive , clinical remission rate at 6 and 12 months was significantly reduced in patients who were ARA+ patients than in ARA− (9/26 [35%] vs. 16/31 [52%], *P* = 0.20 and 14/26 [54%] vs. 27/31 [87%], *P* = 0.008, respectively). Differently, considering only non–rituximab-naive patients, clinical remission rate at 12 months after rituximab therapy was reduced in patients with ARA positivity before a subsequent rituximab course compared with patients who were ARA− (5/9 [56%] vs. 7/8 [88%], respectively; *P* = 0.29), although with no statistical significance. In patients who were ARA+, clinical remission rate at 12 months was comparable in rituximab-naive patients to that of non–naive patients (14/26 [54%] vs. 5/9 [56%], respectively; *P* = 1.0).

The maximal value of ARA titer was not significantly different between patients with and without clinical remission after rituximab therapy (36.2 vs. 36.3 ng/ml, respectively; *P* = 1.00). Moreover, considering a median ARA titer of 25 ng/ml, patients with a maximal ARA titer < 25 ng/ml had a similar clinical remission rate to that of patients with a maximal ARA titer > 25 ng/ml (*P* = 0.88). Taken together, this information demonstrates that ARA titer has no impact on the clinical remission rate. We then analyzed whether ARA development could have an impact on the rate of relapse in patients who reached clinical remission and with at least 12 months of follow-up. The ARA positivity was associated with a significantly higher rate of relapse, being observed in 12 of 19 ARA+ patients (63%) versus 10 of 34 ARA− patients (29%) (*P* = 0.036). Notably, relapse occurred earlier in ARA+ (median: 22 months) than in ARA− patients (median: 32 months) (*P* = 0.01).

### Correlation Between ARA Development and Hypersensitivity Reactions in Patients With pMN

We finally evaluated the incidence of hypersensitivity reactions in our cohort of rituximab-treated patients. Seven patients experienced the following hypersensitivity reactions: skin rash (4), persistent hypotension (1), fever and urticaria (1), and epigastralgia (1). ARA development does not appear to be associated with an increased risk of hypersensitivity reactions (4 patients in ARA+ vs. 3 patients in ARA−).

## Discussion

Rituximab is a first-line treatment for the management of pMN. However, treatment failure may occur in nearly a third of cases.^28^ One of the main factors leading to treatment failure is the immunization against the drug, with the occurrence of ARAs.

In this study, we demonstrated that ARAs are a common concern in patients with pMN because in our cohort, 47% of patients developed ARAs, which is consistent with previous studies.^17^, ^18^, ^19^ For the first time, we have shown that ARAs were significantly associated with a reduced clinical remission rate 6 and 12 months after rituximab therapy in pMN. In a previous article involving only patients from our French cohort^18^ the clinical remission rate was also reduced in ARA+ patients but without reaching statistical significance because of a smaller population size. As previously showed,^18^ ARAs were significantly associated with a higher relapse rate after rituximab therapy. Faster B-cell reconstitution may explain the effect of ARAs on relapse and remission rates. Unlike anti-PLA2R antibody titer,^29^ which correlates with patient prognosis, ARA titer was not associated with clinical outcome.

Based on Figure 1b, it appears that the rate of positivity for ARA progressively increases with exposure to subsequent rituximab courses; we can explain this result by considering that ARA+ patients become resistant to rituximab or relapse earlier and are therefore more likely to repeat rituximab courses. Accordingly, we have previously shown that ARAs occurred after the first rituximab course in most of the patients,^18^ although patients followed-up with over time with serial ARA assessments who tested positive only after the second rituximab course have also been identified rarely in our cohort. Furthermore, to confirm this hypothesis, considering ARA assessments in individual patients, the rate of ARA positivity was not significantly different after the first rituximab course compared with the rate after a second or third course of rituximab (26/57 [46%] vs. 9/17 [53%], *P* = 0.70) (Figure 1a).

For the first time, we have monitored the kinetics of ARAs following rituximab administration. As previously showed,^18^ ARAs were detected after a median of 9 (interquartile range: 6–12) months following rituximab administration. We have shown that in treatment-naive patients, ARAs were not detected until 6 months after rituximab administration, with a peak at around 9 or 12 months (Figures 2 and 4). In non–rituximab-naive patients who have previously developed ARAs before restarting treatment, ARAs disappeared in all patients, and reappeared with variable timing, generally after the third month following rituximab readministration (Figures 3 and 4). In the latter case, there are 2 potential hypotheses as follows: (i) rituximab depletes ARA-producing B cells, or (ii) a limitation of the enzyme-linked immunosorbent assay which does not detect antibodies conjugated to the drug and therefore cannot detect ARAs as long as rituximab is detectable in the serum (usually 3–6 months after infusion).^30^

These results lead us to propose standardizing the monitoring of ARAs at both 9 and 12 months following rituximab infusion, in addition to the monitoring of ARAs before rituximab readministration, because we have shown that it would allow the identification of about 90% of patients with ARAs. In contrast, monitoring ARAs in the first 6 months after rituximab infusion, for example, following a lack of improvement in proteinuria or anti-PLA2R titer, would not identify most cases of ARA+. The earlier the monitoring of ARAs after rituximab infusion, the higher the risk of false negative.

This monitoring of ARAs will allow the identification of mechanisms of resistance to rituximab and the proposal of suitable therapeutic alternatives in order to personalize patient management. In ARA+ patients, therapeutic alternatives such as obinutuzumab or ofatumumab are more efficient.^11^^,^^27^ In fact, ARAs rarely, if ever, cross-react with human or humanized anti-CD20 monoclonal antibodies.^17^^,^^27^^,^^31^

Our study has several limitations. First, it is a retrospective study, and the modest sample size of the study precludes robust conclusions. However, it is a multicenter study and the largest cohort on this topic. Second, a part of our French cohort has already been included in a previous article. However, patient follow-up has been extended by almost 2 years and the topics of the articles are different. Third, although all patients received conservative treatment, this was not standardized.

To conclude, these results highly support the importance of ARA monitoring in patients with pMN. ARAs are common in patients with pMN previously treated with rituximab. The presence of ARAs represents one of the most important prognostic factors in pMN, because it is significantly associated with a lower remission rate and a higher relapse rate after rituximab treatment. This is especially true, also considering that ARAs are common in patients with pMN treated with rituximab. Patients with ARAs require specific therapeutic adjustments with human or humanized anti-CD20 drugs. These results should be confirmed in larger-scale studies.

## Disclosure

All the authors declare no conflicting interests.

## Data Availability Statement

The raw data supporting the conclusions of this article are available from the authors upon request.

## Author Contributions

MA, MT, AV and BSP contributed to the conception and design of the study. MA contributed to the analysis and interpretation of data. All the authors participated in drafting of the article and approved the final version submitted.
